# A Detection Method for Open–Close States of High-Voltage Disconnector in Smoky Environments

**DOI:** 10.3390/s25051280

**Published:** 2025-02-20

**Authors:** Lujia Wang, Yifan Chen, Jianghao Qi, Kai Zhou, Zhijie He, Lei Jin

**Affiliations:** 1 School of Electrical Engineering, China University of Mining and Technology, Xuzhou 221116, China; yifan.chen@cumt.edu.cn (Y.C.); ts23230138p31@cumt.edu.cn (J.Q.); 2 State Grid Hubei Electric Power Research Institute, Wuhan 430074, China; 13476157793@163.com (K.Z.); 18771129266@163.com (L.J.); 3 Zhangzhou Power Supply Company, State Grid Fujian Electric Power Co., Ltd., Zhangzhou 363000, China; 13559663903@163.com

**Keywords:** disconnector, open–close state, LiDAR, point clouds

## Abstract

Computer vision-based state recognition is widely employed in substations, but conventional video monitoring systems often encounter challenges during emergency situations, such as smoke from fires. In such scenarios, LiDAR emerges as an appealing alternative, capable of capturing the depth information of the target. However, when smoke concentration is high, the quality of collected point cloud data deteriorates, impacting the assessment of the disconnector open–close status. This paper delves into the impact of a smoky environment on point cloud data and introduces a two-stage discrimination process. Firstly, a feature extraction method using sliced point clouds is employed to construct edge features of the conductive arm. Building upon this foundation, an open–close position identification method based on edge pre-processing is employed to obtain the final measurement results. Field experiments demonstrate that the proposed method effectively mitigates smoke interference and accurately determines the disconnector’s open–close status with high reliability and precision. This approach could serve as a reference for the development of continuous disconnector closing state monitoring technology.

## 1. Introduction

Unmanned operation is a major feature of intelligent substations, and one of the important issues it faces is the safe and reliable operation of electrical equipment. As one of the most widely used high-voltage switching devices, a high-voltage disconnector primarily operates in outdoor environments [1,2,3]. The effects of environmental factors and prolonged operation can lead to issues such as oxidation and corrosion in its metal transmission mechanism. This, in turn, can result in sticking or jamming during the opening and closing process, causing equipment damage, power outages, or accidents involving personnel [4,5]. Therefore, it is important to monitor the opening and closing status of a high-voltage disconnector.

The current methods for monitoring the status of isolation switches are primarily based on visible light cameras [6,7,8,9]. However, when encountering smoggy weather or situations such as fires generating dense smoke in substations, visible light cameras face difficulty in capturing effective image samples in smoky environments [10,11]. This hinders further image processing, leading to a decrease in the system’s ability to monitor the status of disconnectors. That makes three-dimensional laser scanning technology a new approach to solving the above-mentioned issues. As the physical embodiment of this technology, LiDAR can rapidly acquire the depth information of objects and save it in the form of point cloud data [12,13,14]. Point clouds, compared to pixel images, offer higher potential for secondary development. However, high concentrations of smoke in the environment can have a negative impact on the point cloud data collected by laser radar. Relying solely on manual processing will constrain the convenience and speed of applying LiDAR in the field. The anti-smoke interference processing of point clouds from a disconnector has become a pressing challenge that needs to be addressed in the application of laser radar in the field of isolation switch monitoring.

In recent years, much work has been conducted on the degradation of lidar point cloud data in smoky environments. Kutila et al. tested the performance of 905 nm LiDAR in a fog chamber and showed that when smoke appears in front of the LiDAR [15], the reflected strength of the real target is greatly weakened, and the small target disappears completely in dense fog. Alexander et al. simulated adverse weather conditions in a climate chamber, installing a LiDAR device on a mobile vehicle to measure static obstacles [16]. The results indicate that due to the strong backscatter of fog, targets with low reflectivity form a ring around the laser radar. High-reflectivity targets are partially visible but with much lower intensity values, as most of the reflected light intensity is attenuated due to the scattering of fog. Li et al. quantitatively investigated the relationship between the range performance of TOF lidar and fog [17]. They found that in a smoky environment, higher visibility, higher target reflectivity, and shorter target distances lead to smaller measurement errors. In the visualization results, they observed that as visibility decreases and target distances increase, targets gradually disappear within the detection range. However, targets with higher reflectivity exhibit better robustness in fog. Other studies have shown the same results as the above studies [18,19,20,21,22]. Therefore, interference from smoky environments can lead to a reduction or even loss of the point cloud data density acquired by LiDAR. This poses a challenge for the processing of point clouds from a disconnector and the discrimination of their open–close positions.

In summary, employing three-dimensional laser scanning technology for the non-contact discrimination of the open–close state of a disconnector holds theoretical feasibility. However, there is currently limited research on the application of this technology in smoky environments. With this in mind, in this paper we propose a method to detect the trip position of a disconnector based on 3D laser scanning technology, and the contributions of our work are as follows:

1. Utilizing point cloud data from a disconnector, the impact of smoky environments on the data was analyzed. Subsequently, a two-stage process, designed to align with the structural characteristics of disconnectors, was proposed to address smoke interference.
2. Two methods have been established, one for feature extraction based on sliced point clouds and another for the open–close position identification method based on edge pre-processing. The aforementioned methods can enhance the accuracy of isolator and circuit breaker position determination for laser radar in smoky environments and reduce reliance on point cloud density.
3. A smoke environment simulation experimental platform was established, and the experimental results validated the feasibility and reliability of the methods proposed in this article. This research serves as a reference for the development of disconnector closing status monitoring technology based on LiDAR.

## 2. Methodology

**Figure 1:**
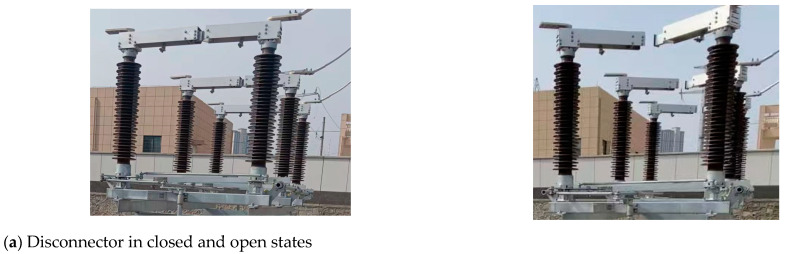

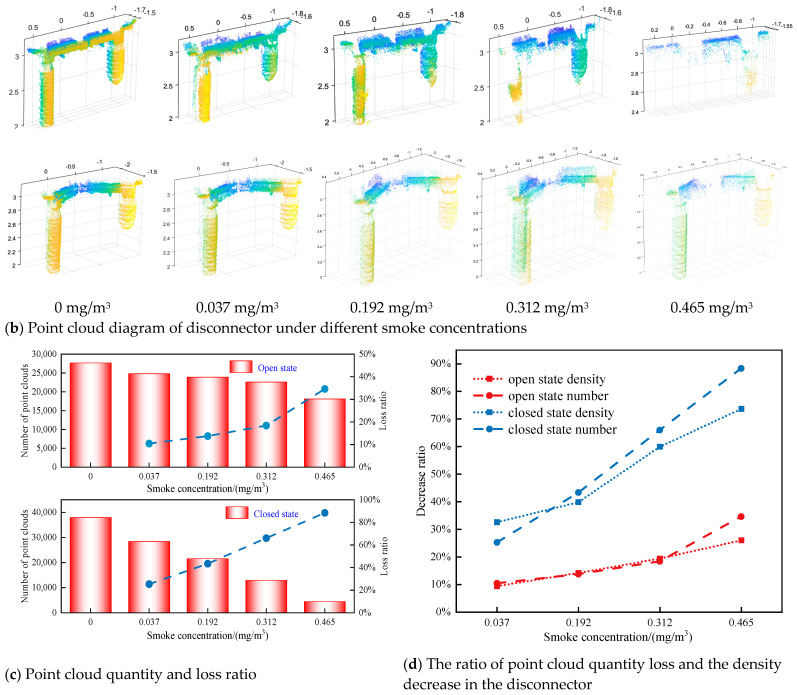
Disconnector point cloud data visualization result.

**Figure 2:**
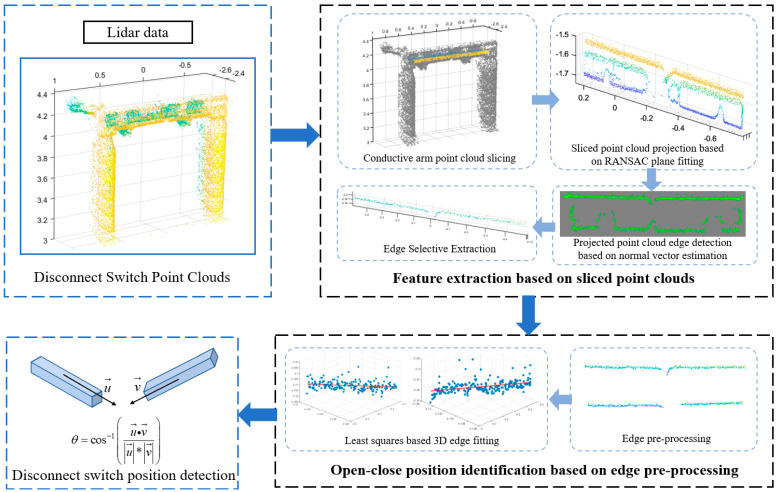
Two-stage discrimination process.

Point cloud data for the disconnector were collected under different concentrations of smoke interference. Two sets of data were obtained, one corresponding to the closed state of the disconnector and the other corresponding to the open state. The collected data were subsequently subjected to visualization analysis, and the results are presented in Figure 1. From Figure 1b, it can be observed that as the smoke concentration increases, the point cloud images of the disconnector gradually become blurred, accompanied by noticeable data loss in the point cloud. To facilitate a more quantitative analysis, the loss ratio of the point cloud quantity and the reduction ratio of the point cloud density for the disconnector were calculated. The point cloud density was determined using the calculation method for LiDAR point cloud density, as expressed by the following equation:

(1) $$\rho =\frac{n-\sum_{i=0}^{m}n_{i}}{A-\sum_{i=0}^{m}A_{i}}$$

where *ρ* is the point cloud density, *n* is the total number of point clouds, *n_i_* is the number of point clouds in the i-th domain, *A* is the sample area, and *A_i_* is the area of the i-th domain. The results of the calculations are presented in Table 1. As shown in the table, with the increase in smoke concentration, the proportion of lost point clouds for the isolating switch also rises, reaching a maximum of 88.38%. Simultaneously, the proportion of the decrease in point cloud density exhibits an upward trend, reaching a maximum of 73.65%. As shown in Figure 1d, the trends of the two indicators exhibit similar patterns under both the opening and closing states of the same isolating switch, with the numerical results being closely aligned. In summary, this indicates that under smoke interference, point cloud data may experience missing values. This is due to the scattering of the laser caused by the presence of smoke particles in the environment. The incident light of the laser is redistributed and scattered in various directions within the space, thereby reducing the intensity of light directed toward the target. Additionally, the laser’s energy diminishes as it propagates, resulting in a decrease in both point cloud quantity and density [23,24]. This, in turn, poses challenges for subsequent point cloud processing. In order to guarantee the precision of the assessment of the open–close positions of the disconnector in the context of incomplete point cloud data, the disconnecting switch tap-changing position recognition method proposed in this paper consists of two steps, as shown in Figure 2, which are feature extraction based on sliced point clouds, and the open–close position identification method based on edge pre-processing, respectively.

### 2.1. Feature Extraction Based on Sliced Point Clouds

*(1) Conductive arm point cloud slicing:* The large size of the conductive arm of the disconnecting switch results in a huge amount of data in this part of the point cloud, and because of its own mechanical structure, there are many bending parts on its surface, which is not conducive to direct fitting and measurement. Since the ultimate purpose of feature extraction is to obtain the spatial direction of the conductive arm, slicing the conductive arm can better solve the above problem, which reduces the amount of data and avoids the mechanical parts that may interfere with the judgment of the switching position.

A point cloud group slicing method based on bubble sorting is used for slicing, in which the point clouds of the conductive arms are first sorted, and the strategy of descending bubble sorting is used according to the *X*-axis coordinate component of each point in the dataset, which facilitates the subsequent slicing of the point clouds. Secondly, the sorted conductive arm point cloud is sliced, and the rule of slicing is to extract a thin slice with a normal vector that is perpendicular to the *yOz* plane along the direction of the descending order of the *X*-axis component of the point cloud, and the expression of its slicing condition is as follows:

(2) $$\left(x_{i},y_{i},z_{i}\right)=\left\{\begin{array}{l} \left(0,0,0\right),\left|x_{i}-x_{j}\right|\geq 10\frac{\sigma l}{L}\\ \left(x_{i},y_{i},z_{i}\right),\left|x_{i}-x_{j}\right|\leq 10\frac{\sigma l}{L} \end{array}\right.$$

where *x_i_*, *y_i_*, *z_i_* are the coordinates of each point in the point cloud; *x_j_* is the *X*-axis component of the slice plane, and the size of its value depends on the number of slices as well as the spacing; *σ* is the error generated when the distance between the LiDAR and the target object is *L*; and *l* is the actual distance between the measurement object and the device. When the distance from a data point to the selected slice plane is less than 10 *σl*/*L*, the point is retained as a point within that slice, otherwise it is discarded. After completing all the slices, they are selected, and the selected slices are required to retain complete edge information for subsequent edge detection and fitting.

*(2) Sliced point cloud projection based on RANSAC plane fitting:* Due to the existence of a certain thickness of the sliced point cloud, in order to reduce the computational difficulty and the amount of computation during edge detection, the sliced point cloud is projected to the plane, which can both reduce the thickness of the sliced point cloud to the minimum and ensure sufficient point cloud density to improve the accuracy of the edge detection and the precision of the edge fitting. Since the plane where the LiDAR transmitter and collector are located is parallel to the *yOz* plane in the point cloud coordinate system, the sliced point cloud obtained after projection to the *yOz* plane is the orthographic projection of the original 3D point cloud to the *yOz* plane.

The data of the plane to be projected needs to be obtained before the plane is projected. The required planar data can be obtained based on the Random Sampling Consistency Algorithm (RANSAC), which uses the idea of finding a model to fit the data by first randomly selecting three points in the initial point cloud and calculating their corresponding planar equations [25]. Calculate the algebraic distance from all points to this plane as follows:

(3) $$d_{i}=\left|Ax_{i}+By_{i}+Cz_{i}+D\right|$$

Secondly, the threshold d*t* is selected, for a point, and if *d_i_* ≤ *d_t_*, the point is taken as the inner point of the target plane, otherwise it is the outer point. Meanwhile, the plane normal vector is selected, and since the *yOz* plane is taken as the plane to be projected in this paper, the normal vector is set to be (1, 0, 0). The above steps are then repeated to select the best fit parameters corresponding to the plane with the highest number of interior points, and at the end of the iteration, the coefficients *A*, *B*, *C*, and *D* of the planar model are output. After obtaining the data for the projection plane, the point cloud can be projected into the plane. The coordinates on the plane to be projected correspond to the coordinates of the original sliced point cloud as follows:

(4) $$\begin{array}{l} x_{p}=x\cdot (B^{2}+C^{2})-\frac{A\cdot (y\cdot B+z\cdot C+D)}{\left.\left\|\left[A,B,C\right]\right.\right\|}\\ y_{p}=y\cdot (A^{2}+C^{2})-\frac{B\cdot (x\cdot A+z\cdot C+D)}{\left.\left\|\left[A,B,C\right]\right.\right\|}\\ z_{p}=z\cdot (A^{2}+B^{2})-\frac{C\cdot (x\cdot A+y\cdot B+D)}{\left.\left\|\left[A,B,C\right]\right.\right\|} \end{array}$$

*(3) Projected point cloud edge detection based on normal vector estimation:* Since the interior of the projected point cloud contains a very large number of extraneous points, which is not conducive to subsequent edge extraction, the edge detection of the sliced point cloud reduces the impact of extraneous points on subsequent processing. According to the feature that the edge of the point cloud is located in the region where the normal vector changes drastically, the edge detection method based on normal vector estimation is adopted here, and the edge points are selected by estimating the normal vector of the tangent plane corresponding to each point and combining with the calculation of the angle of pinch. First, set the search radius *r* for any point P in the data points, and take the neighborhood points within the radius as the set of points of the plane to be fitted (*x_i_*, *y_i_*, *z_i_*), and the equation of the plane to be fitted is as follows:

(5) $$\left\{\begin{array}{l} ax+by+cz=d\\ a^{2}+b^{2}+c^{2}=1 \end{array}\right.$$

The constraints in the above equation are used to limit the size of the normal vector, and the distance *d_i_* from each point to the plane in the projected point cloud can be calculated according to (5), which is given below:

(6) $$d_{i}=\left|ax+by+cz-d\right|$$

The eigenvector corresponding to the smallest hour of solving (7) according to the above equation is, i.e., the normal vector estimated at point P.

(7) $$f=\sum_{i=1}^{n}d_{i}^{2}$$

**Figure 3:**
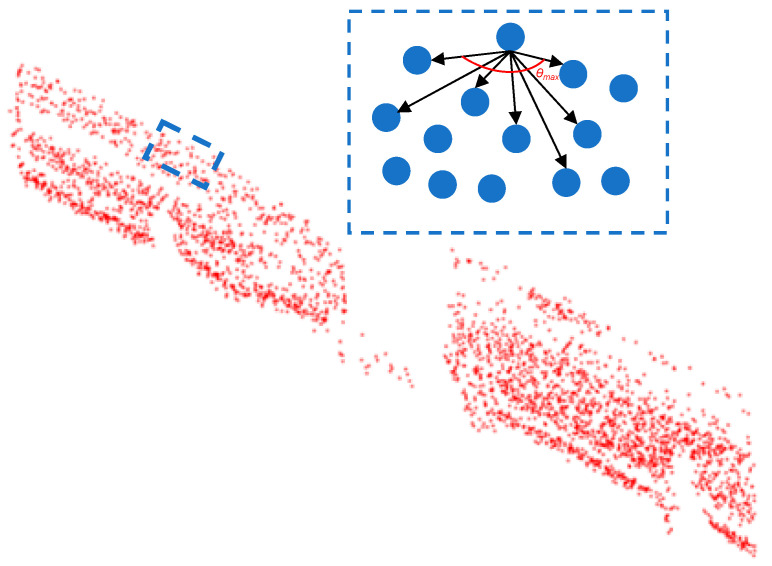
Demonstration of *θ*_max_ selection.

Let the normal vector of point P be *n*, and its corresponding tangent plane is *m*. Project the points in the point cloud onto this plane, select a point counted as P’ from it, and take it as a corner point. Connect the projected neighborhood points with P’ two by two in clockwise order to form a trip to a series of intersection angles *θ_i_* (i = 1, 2, 3…). As illustrated in Figure 3, the maximum angle, *θ*_max_, is identified. When this angle exceeds the specified threshold, the point P is classified as a boundary point. The aforementioned process should be repeated in order to complete the edge detection of the projected point cloud.

*(4) Edge Selective Extraction:* Once edge detection is complete, the upper edge is typically selected as the feature of the guide arm to characterize its spatial orientation. In the absence of smoke interference or a low smoke concentration, the upper edge point cloud is more dense and exhibits superior continuity in space compared to the lower edge. Consequently, it is more suitable for subsequent switching position discrimination. However, as the smoke interference gradually increases, the reflection of the laser from the smoke will cause the echo intensity of the isolation switch to decrease and the point cloud at the upper edge will become sparse, in which case, the lower edge of the sliced point cloud will instead be less affected by the smoke interference and therefore the lower edge should be chosen to characterize the position of the conductive arm when the upper edge is insufficient to be used as a feature.

A fast approximate nearest neighbor search algorithm based on KD-Tree is used to construct a bracket box that can encompass the upper edge, and feature extraction can be completed by searching for the point cloud within the bracket box. In the case where the lower edge is to be extracted as a feature, the sliced point cloud after edge detection is rotated 180° around the normal vector of the *yOz* plane according to (8), and at this time, the lower edge is rotated to the upper part, and the extraction of the lower edge can be completed by the preset bracket box.

(8) $$(X_{i},Y_{i},Z_{i})=(x_{i},y_{i},z_{i})\left[\begin{array}{ccc} 1 & 0 & 0\\ 0 & \cos\theta & \sin\theta \\ 0 & -\sin\theta & \cos\theta \end{array}\right]$$

### 2.2. Open–Close Position Identification Method Based on Edge Pre-Processing

**Figure 4:**
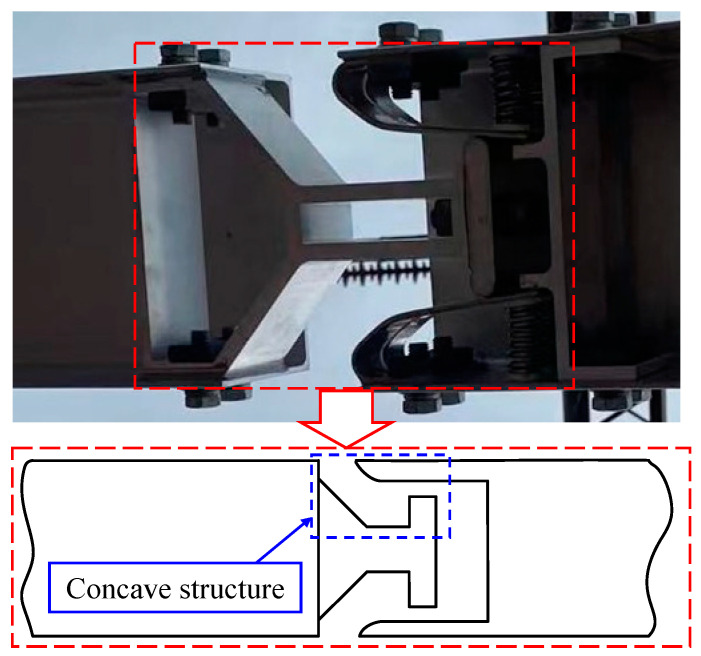
Disconnector contact structure.

**Figure 5:**
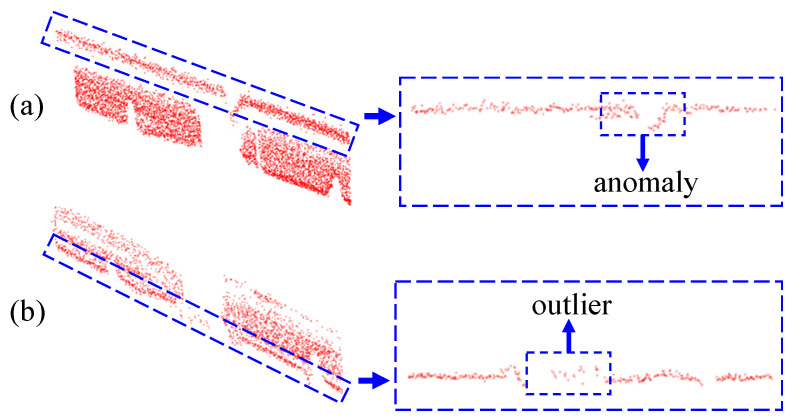
(**a**) Anomalies in the upper edge of the slice point cloud. (**b**) Outliers in the lower edge of a sliced point cloud.

*(1) Removal of the Anomaly on the Upper edge:* In the center of the point cloud at the upper edge, there is a piece of the point cloud that is raised downwards, which is due to the mechanical structure of the conductive arm of the disconnector itself. Figure 4 shows the structure of the contact part of the disconnector, and it can be seen that the contact part has a concave structure, resulting in an unfavorable anomaly for edge fitting at corresponding positions on the upper edge, as shown in Figure 5a.

In order to eliminate outliers, it is imperative to establish both the width of each segment and the width of the overlapping region between consecutive segments. Subsequently, upon ascertaining the upper and lower bounds of the *Y*-axis component within the upper edge point cloud, the number of segmented point clouds can be determined, calculated as follows:

(9) $$count=\frac{y_{\max}-y_{\min}}{w_{\mathrm{seg}}-p_{\mathrm{seg}}}$$

where count is the number of segments, *y*_max_ is the maximum y-coordinate of the point cloud data, *y*_min_ is the minimum y-coordinate of the point cloud data, *w*_seg_ is the width of each segment, and *p*_seg_ is the width of the overlapping region between segments. Secondly, using the index of the segment and the segment parameters, the y-coordinate range of each segment is calculated and the point cloud data within this range is selected and stored in an array. Subsequently, the algorithm traverses the entire dataset, identifying the segment with the lowest lower *Z*-axis boundary, which is subsequently designated as the anomalous segment. Ultimately, the corresponding points are eliminated from the upper edge point cloud in accordance with the index of the returned point cloud for that specific segment.

*(2) Removal of Outliers on the Lower Edge:* Due to missing point cloud data, the anomaly caused by the mechanical structure of the contact part of the conductive arm is no longer obvious, gradually evolving into outliers sandwiched between the point clouds of the left and right conductive arms, as shown in Figure 5b. Therefore, when the lower edge of the sliced point cloud is used as the feature of the conductive arm, the method of setting up an enclosing box to encapsulate the outliers and then deleting them from the original point cloud is used to remove the noise between the edges of the two conductive arms, which is conducive to the subsequent clustering and segmentation to divide the edges into the left and right sub-blocks.

*(3) Least squares-based 3D edge fitting:* The pre-processed edge point cloud should be segmented prior to edge fitting, using the Euclidean clustering segmentation algorithm to separate the left and right sub-blocks and store them in different arrays. The two sub-blocks are then each fitted with 3D straight lines to obtain unit vectors that can characterize their respective 3D spatial directions. First, the index of each point in the X, Y and Z axes of the point cloud data is queried and assigned to the variables *x*_i_, *y*_i_ and *z*_i_, respectively, which are combined into a matrix to form the point cloud data matrix and assigned to the variable *pd*. *pd* is then decentered to make the line pass through the origin during the fitting process, which simplifies the computation process. The coordinates of the center of mass of the point cloud are calculated as follows:

(10) $$cen=\left(\bar{x},\bar{y},\bar{z}\right)=\left(\frac{1}{N}\sum_{i=1}^{N}x_{i},\frac{1}{N}\sum_{i=1}^{N}y_{i},\frac{1}{N}\sum_{i=1}^{N}z_{i}\right)$$

Then, each point’s coordinates are subtracted from the centroid coordinates, and the results are assigned to the de-centered point cloud data matrix *D*. A matrix singular value decomposition is performed on *D* to extract the feature information, including the direction vector of the straight line. In the decomposition result, the singular value corresponding to V is the largest, indicating the direction of the greatest data variation. Therefore, its first column is taken as the direction vector of the spatial line. Simultaneously calculate the coordinates of the centroid (*a*, *b*, *c*). With the direction vector and centroid coordinates, the parameter equation of the spatial line can be obtained. The 3D Edge Fitting algorithm is presented in Algorithm 1. The spatial lines *L*_1_ and *L*_2_ obtained through edge fitting can be used to characterize the spatial orientation of the conductive arms. Based on Equation (11), the angle between the direction vectors of the left and right conductive arms can be calculated, serving as a basis for determining the position of the disconnector.

(11) $$\theta =\cos^{-1}\left(\frac{\vec{u}\cdot \vec{v}}{\left|\vec{u}\right|*\left|\vec{v}\right|}\right)$$

where $\vec{u}$ and $\vec{v}$, respectively, represent the direction vectors of the spatial lines.
**Algorithm 1** 3D Edge Fitting from Pre-Processed Point Clouds**Input:** pre-processed edge point clouds *P***Output:** Spatial position of conductive arms *L*_1_, *L*_2_    Initialize minimum clustering distance *D_m_*    Perform Euclidean clustering: [lab, num] = pcsegdist(*P*, *D_m_*);    **for**
*j* = 1: num **do**    search set of cluste points *P_j_* = {*P_i_*∈*P* | lab[*P_i_*] = = *j*};       (*x*, *y*, *z*) = extractXYZ(*P_j_*);        Constructing point cloud data matrix *pd* ← (*x*, *y*, *z*);        *cen* ← ($\overset{-}{x}$, $\overset{-}{y}$, $\overset{-}{z}$)        *D* ← *pd* − *cen*;        *C* ← (1/(*n*−1))*D*^T^*D*;        *D* ← *USV*^T^;        [*v*_1_,*v*_2_,*v*_3_]^T^ ← [*V*_1,1_,*V*_2,1_,*V*_3,1_]^T^        Calculate center of mass coordinates (*a*, *b*, *c*);        *l_x_* ← *at* + *v*_1_*t*, *l_y_* ← *bt* + *v*_2_*t*, *l_z_* ← *ct* + *v*_3_*t*;    **end for**  **return**
*L*_1_, *L*_2_;

## 3. Tests and Analysis of Results

### 3.1. Construction of Experimental Platforms and Data Acquisition

**Figure 6:**
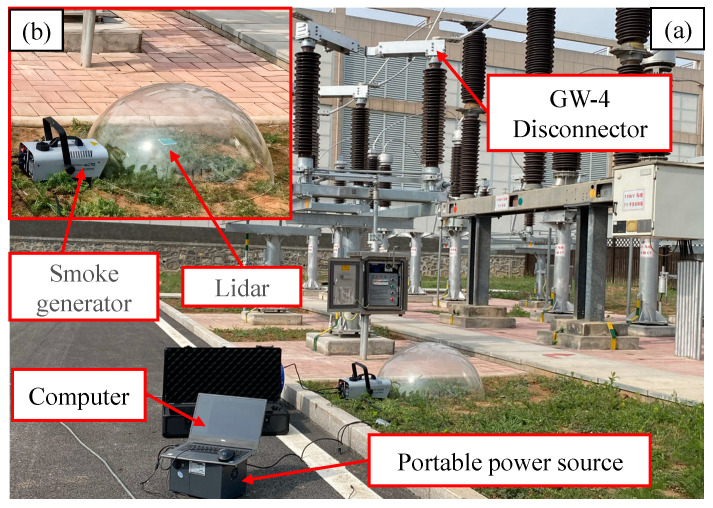
(**a**,**b**) Experimental platform.

In order to verify the feasibility and accuracy of the method, an experimental setup is built in the field, and the GW-4 disconnector is selected as the research object to simulate the point cloud data of the disconnecting switch which is collected and processed under the smoke environment. In the course of the experiment, point clouds were collected using a 905 nm LiDAR. To ensure the requisite safety distance between the equipment, the LiDAR was situated at a horizontal distance of 2 m from the base of the disconnector, with a straight-line distance of approximately 3.8 m from the conductive arm of the disconnector. A smoke generator was utilized to produce smoke, creating a short-term, stable smoke field within an acrylic fog chamber. The smoke particles generated by the smoke generator ranged in size from 5 to 10 μm, effectively simulating the characteristics of various types of fire smoke observed in real-world scenarios [26,27]. The specific device is shown in Figure 6.

**Figure 7:**
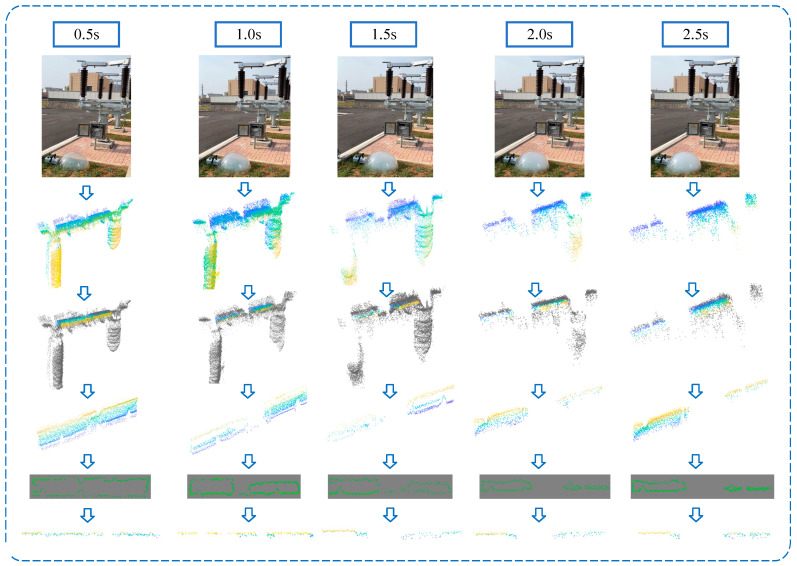
Point cloud data processing results under different smoke concentrations.

Five distinct smoke concentration environments were created through the manipulation of the smoke emission time of the smoke generator. The smoke emission times of 0.5, 1, 1.5, 2, and 2.5 s, respectively, corresponded to real-world smoke concentrations of approximately 0.08, 0.2, 0.32, 0.41, and 0.49 milligrams per cubic meter, respectively. Concurrently, the point cloud data under each smoke concentration are collected when the conductive arm is at five different angles for processing. Taking the disconnector in the fully closed state as an example, the raw point cloud visualization results are shown in Figure 7.

### 3.2. Disconnector Conductive Arm Feature Extraction

According to the method in Section 2, the original point cloud data are processed by slicing, projection, edge detection and edge extraction, and the processing results are shown in Figure 7, respectively, taking the disconnector in the fully closed state as an example.

As can be seen from the results, the sliced point cloud better preserves the edge information of the conductive arm, although the data volume is greatly reduced, and the projection of the sliced point cloud can further compress its “thickness”, which is conducive to the processing of edge detection and edge fitting. Edge detection retains the complete edges of the sliced point cloud and eliminates many irrelevant points, avoiding the easy inclusion of noise when extracting edges.

### 3.3. Open–Close Position Identification of Disconnector

**Figure 8:**
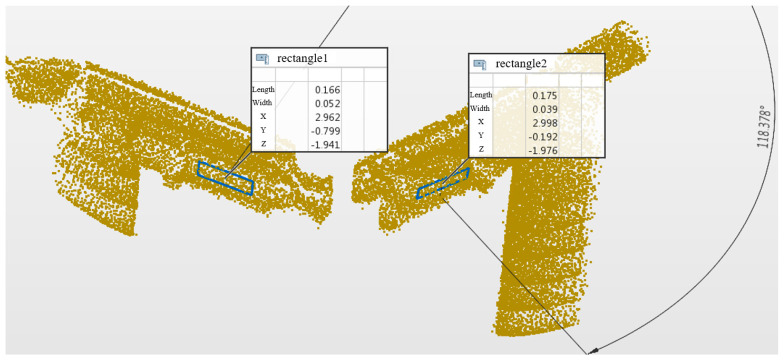
Measurements in PolyWorks.

The extracted feature edges are pre-processed, and then the three-dimensional edge-fitting method is used to measure the angle presented by the two sides of the conductive arm to complete the discrimination of the disconnector open and closed positions. To further validate the accuracy of the measurement algorithm, the raw point cloud data collected in the control group, i.e., when there was no smoke, were imported into the PolyWorks 2022 point cloud measurement software for additional analysis [23,24]. The front surfaces of the left and right conductive arms were fitted, and the normal vectors of the front surfaces were created manually in order to measure the angles presented by the two conductive arms. The results are presented in Figure 8 for the disconnector at the moment of disconnection.

**Figure 9:**
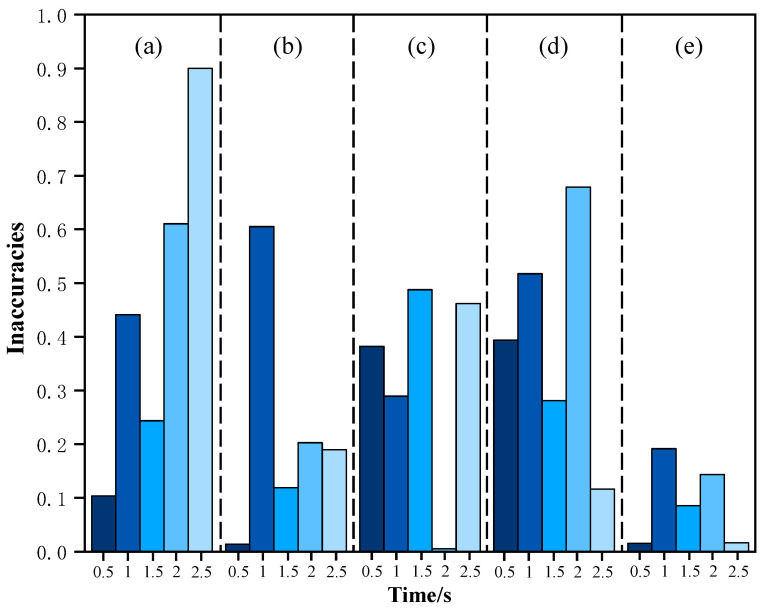
Error rate calculation results. Groups (**a**–**e**) are reference angles of the disconnector conductive arm of 118.3783, 134.9114, 149.8362, 157.7589, and 179.3378, respectively.

The point cloud data collected when there is no smoke in each group are processed according to the method of this paper and the final results are compared with the results obtained from the PolyWorks software as shown in Table 2. From this, it can be seen that the final measurement errors of the two methods can be kept within ±1°, which indicates that the method proposed in this paper is feasible for detecting the switching positions of the disconnector. The point cloud data collected in each group under different smoke concentrations are processed, the results are compared and the error is calculated, and the results are shown in Figure 9, from which it can be seen that the error rate of each group of data is less than 1%. The observed discrepancies primarily arise from differences between the method described in this study and the measurement and calculation process employed by PolyWorks software for determining the relative spatial position of the disconnector’s conductive arm. However, the reliability of PolyWorks has been validated in several studies [23]. Comparisons with PolyWorks results demonstrate that the proposed method achieves high accuracy in identifying the open and closed positions of the disconnector under smoke interference conditions.

## 4. Conclusions

(1) Based on the LiDAR-derived point cloud data, an analysis was conducted to examine the impact of smoke in the environment, revealing that smoke interference leads to partial data loss in the point cloud.
(2) A two-stage discrimination process is proposed. The edge features of the conductive arm are constructed through a feature extraction method based on sliced point clouds. By employing an open–close position identification method based on edge pre-processing, the calculation of the angle of the conductive arm is completed, thereby achieving the discrimination of the open and close states of the disconnector.
(3) Smoke environment simulation experiments were performed to compare the results with manual measurements in PolyWorks. The findings validate that the method proposed in this article can effectively mitigate smoke interference and accurately discern the open and close states of the disconnector, demonstrating its feasibility and reliability.
(4) Although the GW4 isolating switch, the most widely used type in substations, was selected as the validation object in this study, the proposed method for detecting the opening and closing states is also applicable to other isolating switches, such as GW5, GW6, GW16, and GW17 models, which rely on conductive arm movement for operation. In future work, efforts will be made to implement the proposed method across various isolating switch models, enabling the accurate detection of the opening and closing states for switches of different types and voltage levels.

## Figures and Tables

**Table 1:** Point cloud density calculation results.

**Lidar Sensor Specifications**				
Laser wavelength	905 nm	Detection distance	0.5–190 m	
Field of view	70.4° (horizontal) × 77.2° (Vertical)	Angular resolution	0.05° (horizontal) × 0.05° (Vertical)	
Range (100 klx)	190 m/10% reflectivity 230 m/20% reflectivity 320 m/80% reflectivity	Range(0 klx)	190 m/10% reflectivity 190 m/20% reflectivity 450 m/80% reflectivity	
Random error of ranging	<2 cm	Angle random error	<0.05°	
**Point Cloud Quantity Loss Ratio and Point Cloud Density Reduction Rate**				
	Open state		Closed state	
Smoke concentration/(mg/m^3^)	Quantity loss ratio	Density reduction rate	Quantity loss ratio	Density reduction rate
0.037	10.42%	9.41%	25.27%	32.58%
0.192	13.73%	14.19%	43.34%	39.84%
0.312	18.39%	19.39%	65.99%	59.93%
0.465	34.63%	26.02%	88.38%	73.65%

**Table 2:** Measurement results of the proposed methodology and PolyWorks.

Groups	PolyWorks	Methodology of the Paper				
		0.5 s	1 s	1.5 s	2 s	2.5 s
a	118.3783	118.2566	118.9003	118.6666	119.1008	119.4432
b	134.9114	134.8922	134.0945	134.7504	134.6374	134.6551
c	149.8362	149.2635	149.4025	149.1053	149.8284	150.5281
d	157.7589	158.3806	158.5272	157.3154	158.8298	157.5758
e	179.3378	179.3107	178.9945	179.4910	179.5950	179.3081

## Data Availability

Data are contained within the article.
